# Synergistic Detrimental Effects of Cigarette Smoke, Alcohol, and SARS-CoV-2 in COPD Bronchial Epithelial Cells

**DOI:** 10.3390/pathogens12030498

**Published:** 2023-03-22

**Authors:** Abenaya Muralidharan, Christopher D. Bauer, Dawn M. Katafiasz, Heather M. Strah, Aleem Siddique, St Patrick Reid, Kristina L. Bailey, Todd A. Wyatt

**Affiliations:** 1 Department of Pathology and Microbiology, College of Medicine, The University of Nebraska Medical Center, Omaha, NE 68198, USA; 2 Pulmonary, Critical Care, and Sleep Medicine Division, Department of Internal Medicine, College of Medicine, the University of Nebraska Medical Center, Omaha, NE 68198, USA; 3 Department of Surgery, College of Medicine, the University of Nebraska Medical Center, Omaha, NE 68198, USA; 4 Veterans Affairs Nebraska-Western Iowa Health Care System, Omaha, NE 68105, USA; 5 Department of Environmental, Agricultural & Occupational Health, College of Public Health, the University of Nebraska Medical Center, Omaha, NE 68198, USA

**Keywords:** SARS-CoV-2, COPD, alcohol, cigarette smoking, lung injury

## Abstract

Lung conditions such as COPD, as well as risk factors such as alcohol misuse and cigarette smoking, can exacerbate COVID-19 disease severity. Synergistically, these risk factors can have a significant impact on immunity against pathogens. Here, we studied the effect of a short exposure to alcohol and/or cigarette smoke extract (CSE) in vitro on acute SARS-CoV-2 infection of ciliated human bronchial epithelial cells (HBECs) collected from healthy and COPD donors. We observed an increase in viral titer in CSE- or alcohol-treated COPD HBECs compared to untreated COPD HBECs. Furthermore, we treated healthy HBECs accompanied by enhanced lactate dehydrogenase activity, indicating exacerbated injury. Finally, IL-8 secretion was elevated due to the synergistic damage mediated by alcohol, CSE, and SARS-CoV-2 in COPD HBECs. Together, our data suggest that, with pre-existing COPD, short exposure to alcohol or CSE is sufficient to exacerbate SARS-CoV-2 infection and associated injury, impairing lung defences.

## 1. Introduction

More than 16 million Americans are affected by a disease associated with cigarette smoking, including heart disease, cancer, chronic bronchitis, and chronic obstructive pulmonary disease (COPD), with millions more undiagnosed cases estimated to exist [1]. Alcohol overconsumption is linked to cigarette smoking, with more than 80% of people who abuse alcohol also smoking cigarettes [2]. Furthermore, alcohol abuse has been associated with lung diseases such as pneumococcal pneumonia [3] and COPD [4]. Alcohol metabolism can cause severe injury to the respiratory system, including acute respiratory distress syndrome, profoundly impacting the innate and adaptive immunity in the lungs [5]. This can increase the likelihood of pulmonary infections and exacerbate disease severity.

Severe acute respiratory syndrome coronavirus 2 (SARS-CoV-2), a virus that causes coronavirus disease 2019 (COVID-19), has given rise to one of the largest pandemics in history. Spreading to more than 216 countries and territories in less than 8 months, the number of cases continues to increase worldwide, with more than 600 million confirmed cases and over 6.8 million deaths reported to the World Health Organization (WHO) [6] (https://covid19.who.int/ accessed on 21 February 2023). One of the reasons SARS-CoV-2 has such a high transmission rate is that many infected individuals are asymptomatic. However, when symptomatic, the infected individuals present with a wide range of symptoms from fever to pneumonia to acute respiratory distress to multiorgan failure and death [7]. About 15% of patients develop pneumonia. Compared to other common viral infections, SARS-CoV-2 results in a higher number of hospitalizations with a substantial increase in the need for oxygen therapy and ventilatory support [8,9,10,11]. The severity of the symptoms and resulting prognosis most often depend on various risk factors and underlying conditions. Some of the major risk factors that can increase infection-related morbidity and mortality are older age [12], obesity [13], cigarette smoking [14], alcohol use disorders [15], and pre-existing lung disease [15,16].

Smoking is the leading cause of COPD. Along with using harmful substances such as cigarette smoke, alcohol, or any agent that can affect the lung environment, infections with respiratory pathogens can further exacerbate pre-existing COPD. Alcohol abuse, one of the top three lifestyle-related causes of death in the United States, increased during the COVID-19 pandemic, potentially significantly impairing lung immunity [17,18,19]. Together, cigarette smoke, alcohol, and SARS-CoV-2 can harm mucosal immunity in a healthy individual. Compounded with COPD, the outcomes can be catastrophic. Indeed, SARS-CoV-2 infection of COPD patients results in more severe disease leading to worse outcomes than non-COPD patients [20]. COPD and smoking are also associated with poor prognosis in COVID-19 patients [16].

Risk factors, such as cigarette smoking, alcohol misuse, and pre-existing lung diseases like COPD, have been shown to exacerbate SARS-CoV-2-mediated mortality and COVID-19 severity. Therefore, it is important to understand the individual effects and synergistic effects of these agents/diseases on immunity and pathogen clearance. Here, we studied the impact of a short exposure to cigarette smoke and/or alcohol on acute SARS-CoV-2 infection of ciliated primary human bronchial epithelial cells isolated from patients with COPD.

## 2. Materials and Methods

### 2.1. Isolation and Culture of Human Bronchial Epithelial Cells (HBEC)

Deidentified human lungs were accepted from LiveOn Nebraska when they could not be utilized for transplantation in accordance with our IRB-approved protocol (IRB # 318-09-NH). The ‘Healthy’ donors of the lungs used in these experiments were free from chronic lung diseases such as COPD or asthma. None of the donors was a current smoker. They had less than a 20-pack-year history of smoking and did not have a history of alcohol use disorder. Ages ranged from 20–64. The ‘COPD’ donors were collected with the University of Nebraska Medical Center (UNMC) lung transplant registry and biorepository (IRB # 122-16-FB). All COPD donors had end-stage COPD requiring transplantation. Their ages ranged from 54–65. All of them had quit smoking for longer than one year but had a greater than 50-pack per year history of smoking.

HBECs were isolated from 3 healthy donors and 3 COPD donors using an established protocol [21]. First, the large airways from the lungs were dissected and placed in a collagenase solution for 36–48 h. Next, the lumens of the large airways were scraped, and the resulting HBECs were plated in collagen-coated plates in Bronchial Epithelial Grow Media (BEGM) (Lonza, Basel, Switzerland). Subsequently, the HBECs were grown at Air-Liquid Interface (ALI) on inserts using Pneumacult-ALI media (StemCell Technologies, Cambridge, MA, USA) for 28 days until cilia were formed and could be validated for normal beating via Sisson Ammons Video Analysis (Ammons Engineering, Clio, MI, USA) [22].

### 2.2. Virus

SARS-CoV-2 wild-type strain USA-WA1/2020 (BEI Resources, Manassas, VA, USA) was propagated in Calu-3 cells (ATCC: HTB-55). All experiments involving viral infections were conducted at the University of Nebraska Medical Center (UNMC) Biosafety Level 3 (BSL3) facility with Institutional Biosafety Committee approval.

### 2.3. HBEC Treatments and Infection

Primary HBECs were grown at the air-liquid interface, as described above. Once HBECs developed cilia, they were treated with 50 mM ethanol (Decon Labs, King of Prussia, PA, USA) and/or 5% cigarette smoke extract (CSE; [23]) diluted in the basilar ALI media for 1 h at 37 °C and 5% CO_2_. Following incubation, the basal media containing ethanol and/or CSE was removed, and fresh ALI media was added. The cells were then infected with 500 plaque-forming units (PFU) of the SARS-CoV-2 virus diluted in ALI media through the apical side. Following an 18-h infection at 37 °C, the viral inoculum was removed, and the cells were scraped and collected in Buffer AVL with carrier RNA (Qiagen, Hilden, Germany). The basal media was collected and stored at −80 °C for LDH assay and IL-8 ELISA.

### 2.4. RNA Extraction and Quantitative Polymerase Chain Reaction (qPCR)

RNA was isolated from the cells using QIAamp Viral RNA Mini Kit (Qiagen, Hilden, Germany) according to the manufacturer’s instructions. UltraPlex 1-Step ToughMix (QuantaBio, Beverly, MA, USA) was used along with 2019-nCoV CDC Probe and Primer Kit for SARS-CoV-2 (Catalog: KIT-nCoV-PP1-1000) for the CoV-2 qPCR reactions. Human ACE-2 primer/probe and 18S Ribosomal RNA control (Applied Biosystems, Waltham, MA, USA) were used for the hACE-2 qPCR reactions. QuantStudio 3 Real-Time PCR machine (Applied Biosystems, Waltham, MA, USA) was used with QuantStudio Design and Analysis software version 1.5.1 (Applied Biosystems, Waltham, MA, USA) for analysis. Results are expressed as CoV-2 or ACE-2 expression determined using 2^−(ΔCt)^ method with 18S ribosome as the endogenous control.

### 2.5. Lactate Dehydrogenase (LDH) Activity Assay

LDH activity was determined in the treated/infected HBEC basal media using an LDH Activity Assay Kit (Sigma-Aldrich, St. Louis, MO, USA) according to the manufacturer’s instructions. LDH activity is reported as milliunit/mL.

### 2.6. Interleukin-8 (IL-8) ELISA

Secreted IL-8 in the treated/infected HBEC basal media was determined using Human IL-8/CXCL8 DuoSet ELISA kit (R&D Systems, Minneapolis, MN, USA) according to the manufacturer’s instructions. IL-8 is reported as ng/mL.

### 2.7. Statistical Analysis

Statistical analysis was conducted using a two-way analysis of variance (ANOVA). Tukey’s post hoc test was used to adjust for multiple comparisons between different test groups. Tests were performed at a 5% significance level. All statistical analyses were performed using GraphPad Prism 8 (San Diego, CA, USA) software.

## 3. Results

### 3.1. Short Exposure to Cigarette Smoke or Alcohol Augments SARS-CoV-2 Infection in HBECs Isolated from COPD Patients

We aimed to determine the effect of ethanol and cigarette smoke on SARS-CoV-2 infection of HBECs isolated from healthy and COPD patients. We exposed fully ciliated healthy and COPD HBECs to ethanol, CSE, or ethanol and CSE combined for 1 h before infecting the cells with SARS-CoV-2. Following infection, the cells were collected using qPCR to determine viral titer and ACE-2 expression. Because HBECs are grown for 3–4 weeks on ALI for proper cilia development, and the number of cells may vary from well to well, the viral titer and ACE-2 levels were normalized to 18S ribosomal endogenous control.

**Figure 1:**
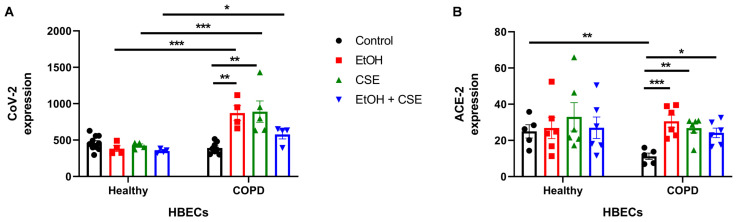
Short exposure to cigarette smoke or alcohol augments SARS-CoV-2 infection in HBECs isolated from COPD patients. HBECs isolated from healthy and COPD donors were grown on an air-liquid interface and ciliated before treatment with 5% cigarette smoke extract (CSE) and/or 50 mM ethanol (EtOH) for 1 h. The treated cells were then infected with 500 PFU of wild-type SARS-CoV-2 for 18 h and collected to determine (**A**) viral titer and (**B**) human ACE-2 expression using qPCR. CoV-2 and ACE-2 expressions shown on the y-axis were determined using the 2^−(ΔCt)^ method with 18S ribosome as the endogenous control. Data shown are mean ± SEM; *n* = 6 (from three healthy donors and three COPD donors) per group in each experiment; experiments were repeated two times; * *p* < 0.05, ** *p* < 0.01, *** *p* < 0.001 (two-way ANOVA with Tukey’s *post hoc* test).

In the healthy HBECs, neither ethanol nor CSE increased viral load, while single treatment of COPD HBECs with ethanol or CSE significantly augmented SARS-CoV-2 titers in the cells compared to the respective ‘no treatment’ controls (Figure 1A). Importantly, single, and combined treatment with ethanol and CSE significantly increased viral load in COPD HBECs compared to the healthy group, whereas viral titer was comparable in the ‘no treatment controls between normal and COPD HBECs. Together, short exposure to alcohol or cigarette smoke compounded with COPD substantially exacerbated SARS-CoV-2 infection in vitro.

We then sought to identify if the changes in viral titer were due to alterations in ACE-2 expression, a SARS-CoV-2 entry receptor. Similar to the pattern observed with infection (Figure 1A), there were no differences in ACE-2 expression within the healthy HBECs, while single treatment with ethanol or CSE significantly increased ACE-2 levels in COPD HBECs compared to the ‘no treatment’ control (Figure 1B). However, with the dual treatment of ethanol and CSE, although ACE-2 levels increased in the COPD group, only a slight (not statistically significant) rise in infection was observed compared to the COPD control group. Interestingly, ACE-2 expression in untreated COPD controls was lower than in untreated healthy controls (Figure 1B), but this did not translate to decreased viral load (Figure 1A). This could be due to alternate mechanisms of entry used by the virus.

### 3.2. SARS-CoV-2 Infection following Short Exposure to Cigarette Smoke and Alcohol Exacerbates Injury in HBECs from COPD Patients but Not in Healthy Patients

**Figure 2:**
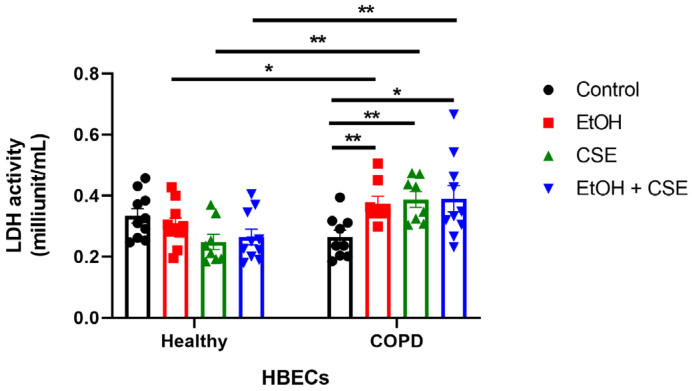
SARS-CoV-2 infection following short exposure to cigarette smoke and alcohol exacerbates injury in HBECs from COPD patients but not in healthy patients. Basal media from treated and infected HBECs were collected 18 h post-infection to determine LDH activity. Data shown are mean ± SEM; *n* = 12 (from three healthy donors and three COPD donors) per group; combined data from two separate experiments are shown; * *p* < 0.05, ** *p* < 0.01 (two-way ANOVA with Tukey’s *post hoc* test).

Following SARS-CoV-2 infection of ethanol/CSE-treated HBECs, we collected the basal media to quantify LDH activity to help determine levels of injury induced by the combination of treatments and viruses in healthy and COPD HBECs (Figure 2). Notably, the trends in LDH activity were similar to that of infection in Figure 1A, with increasing infection levels correlating to higher injury. In the absence of COPD, neither ethanol nor CSE affected LDH activity, while COPD significantly exacerbated ethanol and/or CSE-mediated injury (Figure 2). Moreover, when the only virus was present (‘no treatment’ control), levels of cellular damage in healthy and COPD HBECs were comparable. Therefore, acute SARS-CoV-2 infection in HBECs from donors with COPD following short exposure to alcohol and/or cigarettes results in substantially higher cellular injury compared to HBECs from normal donors.

It is important to note other studies have shown that HBECs exposed to 20% CSE for 48 h or 5% CSE for 24 h do not have an increase in LDH [24,25,26,27,28]. In addition, we have shown in many of our previous studies that 100 mM ethanol does not increase LDH [29,30,31,32,33]. Therefore, since we exposed the cells to 5% CSE and/or 50 mM ethanol for 1 h in this study, we do not expect the treatments alone in the absence of the virus to have any effect on LDH activity in HBECs.

### 3.3. Elevated IL-8 Secretion Correlated to the Synergistic Damage Induced by SARS-CoV-2, Cigarette Smoke, and Alcohol in COPD HBECs

IL-8 is a cytokine that is elevated in the airway epithelium of patients with COPD [34]. Indeed, airway epithelial cells isolated from COPD patients produce more IL-8 at baseline than cells derived from healthy controls [35,36,37,38,39,40,41]. Similarly, it is well-established that CSE increases IL-8 production in transformed airway epithelial cell lines [42,43,44,45] and primary airway epithelial cells [46,47,48,49,50].

**Figure 3:**
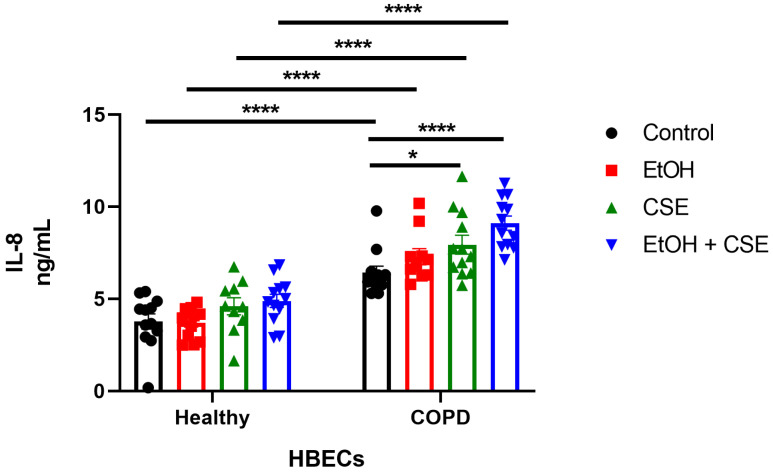
Elevated IL-8 secretion correlated to the synergistic damage induced by SARS-CoV-2, cigarette smoke, and alcohol in COPD HBECs. Using ELISA, basal media from treated and infected HBECs were collected 18 h post-infection to determine secreted IL-8 levels. Data shown are mean ± SEM; *n* = 12 (from three healthy donors and three COPD donors) per group; combined data from two separate experiments are shown; * *p* < 0.05, **** *p* < 0.0001 (two-way ANOVA with Tukey’s *post hoc* test).

As such, we aimed to determine IL-8 release in ethanol/CSE-treated and SARS-CoV-2-infected healthy and COPD HBECs. Therefore, we collected the basal media from treated/infected HBECs and quantified IL-8 secretion using ELISA. In agreement with infection (Figure 1A) and injury (Figure 2), infection of ethanol and/or CSE-treated healthy HBECs had no effect on IL-8 secretion (Figure 3). Furthermore, as expected, IL-8 levels in the ‘no treatment controls of COPD HBECs were significantly higher than that of healthy HBECs. This IL-8 secretion was further augmented with the treatment of CSE alone and a combination of ethanol and CSE in COPD HBECs (Figure 3). Overall, IL-8 levels correlated to the synergistic injury caused by SARS-CoV-2 infection and ethanol/CSE treatment of COPD HBECs.

## 4. Discussion

Individuals who regularly smoke cigarettes are more likely to overconsume alcohol [51], while most individuals with alcohol use disorders also smoke cigarettes [52]. To make matters worse, alcohol use rose during the COVID-19 pandemic [53,54]. Importantly, both cigarette smoke and alcohol can severely compromise the innate immunity of the respiratory system. Consequently, cigarette smoking, alcohol use disorders, and pre-existing lung diseases such as COPD are major risk factors augmenting SARS-CoV-2-related morbidity and mortality [14,15,16,55].

Patients hospitalized for COVID-19 between March and May 2020 with COPD had more severe disease and worse prognoses than non-COPD patients [20]. COPD increased mortality by 1.5-fold in patients with pneumonia, where SARS-CoV-2 was the etiological agent [56]. Furthermore, patients with COPD and COVID-19 pneumonia had more cardiovascular events, longer hospital stays, and a 7-fold increase in mortality compared to non-COVID-19 pneumonia [56].

Alcohol can suppress cough and impair mucociliary clearance from the lung, abrogating innate immunity [57,58]. Furthermore, alcohol abuse has been shown to induce ineffective pathogen clearance and alter inflammatory responses in the lungs [5]. Chronic alcohol use can also increase oxidative injury in the lungs, resulting in elevated inflammatory cytokine levels [59]. For instance, higher levels of the chemokine RANTES and cytokines IL-6 and IL-8 were observed in the bronchoalveolar lavage fluid (BALF) of individuals with alcohol use disorders, predisposing heavy drinkers to more severe COVID-19 [60,61]. Alcohol use disorders increase the likelihood and severity of lung infections in patients with chronic lung diseases such as bronchitis, pneumonia, and COPD [62,63]. COPD and alcohol use together can cause considerable damage to lung immunity, specifically against viral infections. In our study, we showed that an increase in IL-8 secretion (Figure 3) accompanied the augmented infection (Figure 1A) and injury (Figure 2) seen in COPD HBECs following short exposure to alcohol/CSE and acute SARS-CoV-2 infection.

Although some studies at the beginning of the COVID-19 pandemic suggested that nicotine had a protective role against SARS-CoV-2 [64,65], this has been disproven by other recent studies where smoking has been strongly associated with COVID-19 disease severity [66,67,68]. A recent study found increased soluble ACE-2 activity in the BALF of smokers and vapers compared to non-smokers [69]. ACE-2, an entry receptor for SARS-CoV-2, is expressed in membrane-bound and soluble forms in the lungs. Soluble ACE-2 can also bind the spike protein of SARS-CoV-2 and allow viral entry through receptor-mediated endocytosis [70]. Similarly, ciliated HBECs acutely exposed to cigarette smoke (14 puffs of smoke for 1 day) also had elevated membrane-bound and soluble ACE-2 activity compared to air-exposed controls. This translated to increased infection by SARS-CoV-2 pseudovirus in HBECs exposed to cigarette smoke [69]. Here, we used a 1-h ethanol/CSE exposure HBEC model, which was insufficient to see changes in live wild-type SARS-CoV-2 infection in healthy HBECs but enough to increase infection in treated COPD HBECs (Figure 1A).

In contrast to our study, where the cells were infected for one day, single-cell RNA sequencing of differentiated HBECs from healthy and COPD patients 7 days after SARS-CoV-2 infection showed substantially higher infection in COPD HBECs than healthy cells [71]. This correlated with an increase in transmembrane serine protease 2 (TMPRSS2) and cathepsin B (CTSB), proteases involved in SARS-CoV-2 infection, and a decrease in protease inhibitors in COPD HBECs. In addition, inflammatory cytokines such as IL-6, commonly associated with COPD exacerbations and severe COVID-19, were upregulated in COPD HBECs from day 3 post-infection, while interferon responses, specifically IFN-β, were almost completely absent from day 4 post-infection in COPD compared to healthy cells [71]. Inhibition of proteases and therapeutic correction of inflammatory imbalances effectively restored viral clearance in COPD HBECs, highlighting the predisposition of COPD patients to severe COVID-19 [71].

One of the limitations of our study is the lack of multi-cell or in vivo systems where the increase in IL-8 secretion would have resulted in further enhancement of lung injury due to the recruitment of inflammatory cells such as neutrophils. However, primary ciliated HBECs, used in this study, are an excellent, translatable model for human SARS-CoV-2 infection. Indeed, SARS-CoV-2 was detected within multi-ciliated epithelial cells during the early stages of infection in COVID-19 patients, with ACE-2 predominantly localizing to the motile cilia of airway epithelial cells [72,73].

Other studies have established the serious implications of cigarette smoking, alcohol abuse, and lung conditions like COPD on the outcomes of SARS-CoV-2 infection. Together, the effects of these agents/diseases on lung defenses can be further deleterious. Importantly, even a short exposure to alcohol and/or CSE when predisposed to COPD is sufficient to exacerbate SARS-CoV-2 infection, lung injury, and inflammatory responses. When healthy, much longer exposures are needed to impair lung defenses against infection. Therefore, it is vital to understand the individual and synergistic effects of agents/diseases on lung immunity and the effects of the duration of exposure.

## Data Availability

The data presented in this study are available on request from the corresponding author. The data are not publicly available due to privacy concerns.
